# Genome wide-scale CRISPR-Cas9 knockout screens identify a fitness score for optimized risk stratification in colorectal cancer

**DOI:** 10.1186/s12967-024-05323-3

**Published:** 2024-06-10

**Authors:** Xiangchou Yang, Jieyu Liu, Shuaibin Wang, Wail Hussein Ahmed Al-Ameer, Jingting Ji, Jiaqi Cao, Hassan Mansour S Dhaen, Ying Lin, Yangyang Zhou, Chenguo Zheng

**Affiliations:** 1 Department of Hematology and Medical Oncology, The Second Affiliated Hospital & Yuying Children’s Hospital of Wenzhou Medical University, Wenzhou, China; 2 Department of coloproctology, The Second Affiliated Hospital & Yuying Children’s Hospital of Wenzhou Medical University, Wenzhou, China; 3 Department of Urology, The Second Affiliated Hospital & Yuying Children’s Hospital of Wenzhou Medical University, Wenzhou, China; 4 Department of Infectious Disease, The Second Affiliated Hospital & Yuying Children’s Hospital of Wenzhou Medical University, Wenzhou, China; 5 Department of oncology, Shanghai Sixth People’s Hospital, Shanghai Jiao Tong University School of Medicine, Shanghai, 200233 China

**Keywords:** Colorectal cancer, Risk stratification, Fitness genes, CRISPR-Cas9, Cancer-specific fitness score

## Abstract

**Background:**

The molecular complexity of colorectal cancer poses a significant challenge to the clinical implementation of accurate risk stratification. There is still an urgent need to find better biomarkers to enhance established risk stratification and guide risk-adapted treatment decisions.

**Methods:**

we systematically analyzed cancer dependencies of 17 colorectal cancer cells and 513 other cancer cells based on genome-scale CRISPR-Cas9 knockout screens to identify colorectal cancer-specific fitness genes. A regression model was built using colorectal cancer-specific fitness genes, which was validated in other three independent cohorts. 30 published gene expression signatures were also retrieved.

**Findings:**

We defined a total of 1828 genes that were colorectal cancer-specific fitness genes and identified a 22 colorectal cancer-specific fitness gene (CFG22) score. A high CFG22 score represented unfavorable recurrence and mortality rates, which was validated in three independent cohorts. Combined with age, and TNM stage, the CFG22 model can provide guidance for the prognosis of colorectal cancer patients. Analysis of genomic abnormalities and infiltrating immune cells in the CFG22 risk stratification revealed molecular pathological difference between the subgroups. Besides, drug analysis found that CFG22 high patients were more sensitive to clofibrate.

**Interpretation:**

The CFG22 model provided a powerful auxiliary prediction tool for identifying colorectal cancer patients with high recurrence risk and poor prognosis, optimizing precise treatment and improving clinical efficacy.

**Supplementary Information:**

The online version contains supplementary material available at 10.1186/s12967-024-05323-3.

## Introduction

Colorectal cancer (CRC) is a common malignant tumor in the digestive system, originating from the colon and rectal mucosa. According to the global cancer statistics in 2020, of which the incidence rate of colorectal cancer ranks third in the cancers, accounting for 10% of all new cancers; its mortality rate ranks second only behind lung cancer, accounting for 9.4%. It is estimated that by 2030, there will be approximately 2.2 million new cases of colorectal cancer and 1.1 million deaths from colorectal cancer worldwide [1]. Countries with high incidence of CRC are mainly the United States and China, and their prognosis is closely related to TNM staging at the time of onset. The 5-year survival rate of stage IV colorectal cancer patients is less than 8% [2]. Therefore, early screening, diagnosis, and treatment are key to reducing the mortality rate and improving the efficacy of colorectal cancer. Traditional screening methods include endoscopic examination, imaging examination, enterography, and chemical testing, which either have low specificity or insufficient sensitivity [3]. In addition, the occurrence of drug resistance during the treatment process is the main cause of treatment failure, and the emergence of drug resistance genes is the main mechanism of drug resistance occurrence [4]. Therefore, to improve the currently poor clinical prognosis of colorectal cancer, it is essential to involved in its pathogenesis and develop strategies to overcome drug resistance.

Risk stratification refers to accurately predicting the efficacy and prognosis of patients based on individual risk factors, providing personalized treatment plans, and better planning auxiliary treatment and follow-up management by patients, which has important clinical significance [5]. At present, for solid tumor patients, tumor node metastasis (TNM) staging is the main means of survival risk stratification and an important indicator for doctors to judge the patient’s survival risk. However, TNM staging requires pathological testing, which may pose a risk of infection to patients. Colonoscopy is the gold standard for the diagnosis of colorectal cancer, an invasive procedure. On the other hand, China’s limited resources for colonoscopy are frequently utilized by low-risk populations for colorectal cancer. Due to the lack of good stratified screening for colorectal cancer, the effectiveness of colonoscopy in detecting colorectal cancer is limited, and it also wastes resources for colorectal cancer detection, which will increase the economic burden on the population [6, 7]. Therefore, there is an urgent need to develop new risk stratification methods for colorectal cancer patients with high accuracy, good reproducibility, and a simple method to guide the necessity of risk adaptation therapy.

In recent years, extensive research has explored the prognostic factors of CRC patients. Zhao et al. have demonstrated that a colorectal tumor risk stratification model based on fecal immunohistochemistry (FIT) test results and the National Colorectal Polyp Care Program (NCPC) score effectively distinguishes high-risk colorectal cancer populations, thereby improving the efficiency of colorectal cancer screening [8]. This risk stratification model for colorectal cancer can save nearly 50% of colonoscopy examinations and has high sensitivity to the development of colorectal cancer at different stages. This will effectively improve the screening efficiency of colonoscopy in China and broaden the screening coverage for colorectal cancer. Katipallyd et al. established a new classification of liver metastasis in colorectal cancer and substantiated its prognostic importance in the New EPOC trial [9]. The molecular subtypes and clinical molecular risk stratification of liver metastasis in colorectal cancer hold predictive value for patient outcomes, and can also be used as a classification framework widely applicable to other cancers, for developing biomarkers that affect local and systemic treatment of metastatic diseases, identifying patients at the highest risk of relapse, and optimizing individualized treatment plans. The results of Mo et al’s study and other independent cohort studies clearly position ctDNA based molecular residual diseases as the most significant risk factor for recurrence of stage I-II CRC, and are associated with tumor staging and other classic clinical diseases [10].

CRC patients are patients with a wide range of prognostic outcomes. Although risk stratification has multiple potential benefits in CRC, there is still a shortage of colonoscopy resources and low efficiency of colonoscopy examinations [11]. Therefore, a large sample, multicenter, prospective study integrating imaging, genetic, and immunological data is needed to evaluate individual factors in multivariate models, conduct external validation, and develop models. Comparing the performance of different models and developing a CRC risk prediction model tailored for Chinese patients holds significant clinical guidance value for achieving personalized medical care for CRC patients and improving overall survival rates.

The Dependency Map (DepMap) database serves as a valuable resource for exploring cancer treatment targets [12, 13]. This database incorporates data from several collaborators, including the Sanger Research Institute and Novartis. DepMap builds on the original Cancer Cell Line Encyclopedia (CCLE) project, which comprises gene dependency data for over 700 human tumor cell lines derived from various tissue types, along with gene expression, copy number, and mutation information [14]. In DepMap, researchers have performed genome-wide RNAi and CRISPR loss-of-function screens in more than 1000 cancer cell lines to identify the genes required for cell growth. The DepMap database provides insights into the dependency of different cell lines on specific genes. The Broad Institute initially assessed gene dependency using RNAi technology, but CRISPR-Cas9 technology now stands as its primary tool for investigating gene dependency [15]. In parallel, they have employed a multiplexed approach (PRISM) to profile hundreds of cellular models for drug sensitivities. The relationship between genetic dependence, drug sensitivity, and cellular characteristics is determined [16]. We can discover new cancer vulnerabilities, identify biomarkers of drug response, and gain insight into mechanisms of action [17]. The establishment of this database holds significant importance for the advancement of cancer treatment target research [18, 19].

In this study, we systematically analyzed key cancer dependencies of CRC based on genome-scale CRISPR-Cas9 knockout screens. We identified CRC-specific fitness genes and CFG22 score model, which was validated in other CRC cohorts. We also studied the mutational landscape and biological characteristics associated with the CFG22 model score, demonstrating the role of the score in terms of therapeutic response to clofibrate drug.

## Methods

### Source of data

GSE39582 (*n* = 579) [20], GSE17536 (*n* = 145) [21], and GSE161158 (*n* = 200) [22] cohorts of CRC were from Gene Expression Omnibus (GEO). The Cancer Genome Atlas Program (TCGA) of CRC (*n* = 393) was downloaded from https://portal.gdc.cancer.gov/. A total of 1317 patients were enrolled according to the following criteria: (1): Primary colorectal cancer; (2): gene expression profiles and clinical information are available; (3) No chemotherapy or radiotherapy was given before surgery. The GSE39582 cohort was training cohort, and GSE17536, GSE161158, and TCGA of CRC cohorts were validation cohorts. Please refer to Table S1 for the clinical information and sequencing platform of the patients. For TCGA cohort, the RNA-seq raw read count from the TCGA portal is converted into transcripts of millions per kilocase (TPM) and further log_2_(TPM + 1) conversion. For GEO cohorts, data were all retrieved from the Affymetrix GPL570 platform (Human Genome U133 Plus 2.0 Array). Affymetrix’s raw data was processed using the Robust Multiarray Averaging (RMA) algorithm realized in the Affy package. The removal of batch effects from non-biological technical deviations was achieved through the ComBat algorithm in the sva package. Data of Gene effect of each gene for cancer cells was obtained from DepMap. DepMap is to make discoveries related to cancer vulnerabilities by providing open access to key cancer dependencies analytical and visualization tools, which contain the Achilles Project based on genome-scale CRISPR-Cas9 knockout screens [23]. The gene effect reflects the dependence of cancer cells on genes. The lower the gene effect score, the more likely the cell is to rely on the gene.

### Construction of CFG22 score model

We downloaded CRISPR gene effect from DepMap portal. Because some cells lacked information, we selected 17 CRC cells and 513 other cancer other type cells with detailed information of organ origin. Compared with other cancer cell types, 1828 genes of CRC cells that met the conditions of gene effect (CRC cells) < gene effect (other cancer cell types), with p value < 0.1. Then, by univariate analysis, there were 113 genes meeting the condition of hazard ratio > 1, with p value < 0.05. These 113 genes were served as candidate genes in the Least absolute shrinkage and selection operator (LASSO) model. The CFG22 score model contained 22 genes. CFG22 score=-0.171*ATOH1-0.159*CDX2+0.182*CORO2B+0.215*CYBRD1-0.238* DBF4+0.136*DCBLD2+0.169*EGR2+0.151*FAM155A-0.235*FUT7-0.288*GLIS2+0.270*HIVEP2+0.236*HMMR+0.258*HTR2C-0.300*LAMB2-0.278*MEDAG-0.540*NUP37+0.261*PEAR1-0.270*PKD2+ 0.459*PTPN14+0.248*SNAI1+0.133*TREML2-0.225*UBE2E2.

The gene name in the formula represents its expression. According to the median value, we divided patients of each cohort into two groups-CFG22^high^ (CFG22 score ≥ median value) and CFG22^low^ group (CFG22 score < median value).

### Collection of published signatures

We collected 30 signatures from published literature (Table S2). They were all mRNA signatures, which were fitted by lasso algorithms. Risk score = Ʃ (βi * Exp.i) (i = the number of prognostic genes, βi represents the coefficient of gene i, and Exp.i represents expression level of gene i). For CFG22 score model and 30 published signatures, we performed univariate Cox regression and Receiver Operating Characteristic (ROC) analysis.

### Long-term proliferation assay

CRC cells were counted 20,000–50,000 / well (2 ml/ well) into the six-well plate, incubating in the incubator for 24 h. After the cells were attached to the wall, the six-well plate was slowly tilted sideways, and clofibrate was added into the medium according to the gradients of 0 µM, 100 µM, and 200 µM. The medium is changed twice a week and the corresponding concentration of drugs is supplemented. After 10–14 days, the cells were fixed with 4% paraformaldehyde for 10 min, stained with 0.1% crystal violet and allowed to dry.

### Statistical analysis

All statistical tests were performed in R statistical software (Version 4.3.1). Kaplan–Meier evaluation of overall survival and disease free survival and the log-rank test was applied to determine the statistical significance of differences. The hazard ratio was calculated using univariate or multivariate cox regression model using ‘survival’ R package. Time-dependent receiver operator characteristic (ROC) analysis for predicting survival was estimated by ‘timeROC’ R package. The waterfalls map was implemented by ‘maftools’ R package. The nomogram was realized by ‘regplot’ and ‘rms’ R package. Drug prediction was conducted by optimal strategy for signature-based drug repositioning, which was based on Library of Integrated Network-based Cellular Signatures (LINCS) [24]. Gene set enrichment analysis was realized using annotated gene sets of h.all.v2023.1.Hs.entrez.gmt and c5.go.bp.v2023.1.Hs.entrez.gmt, which was performed by ‘HPO.db’ and ‘enrichplot’ R package. Analysis of correlation between two continuous variables was conducted by Pearson’s r correlation.

## Results

### Cancer dependencies analysis identified a 22-gene CRC fitness (CFG22) score correlated with patient survival

**Fig. 1:**
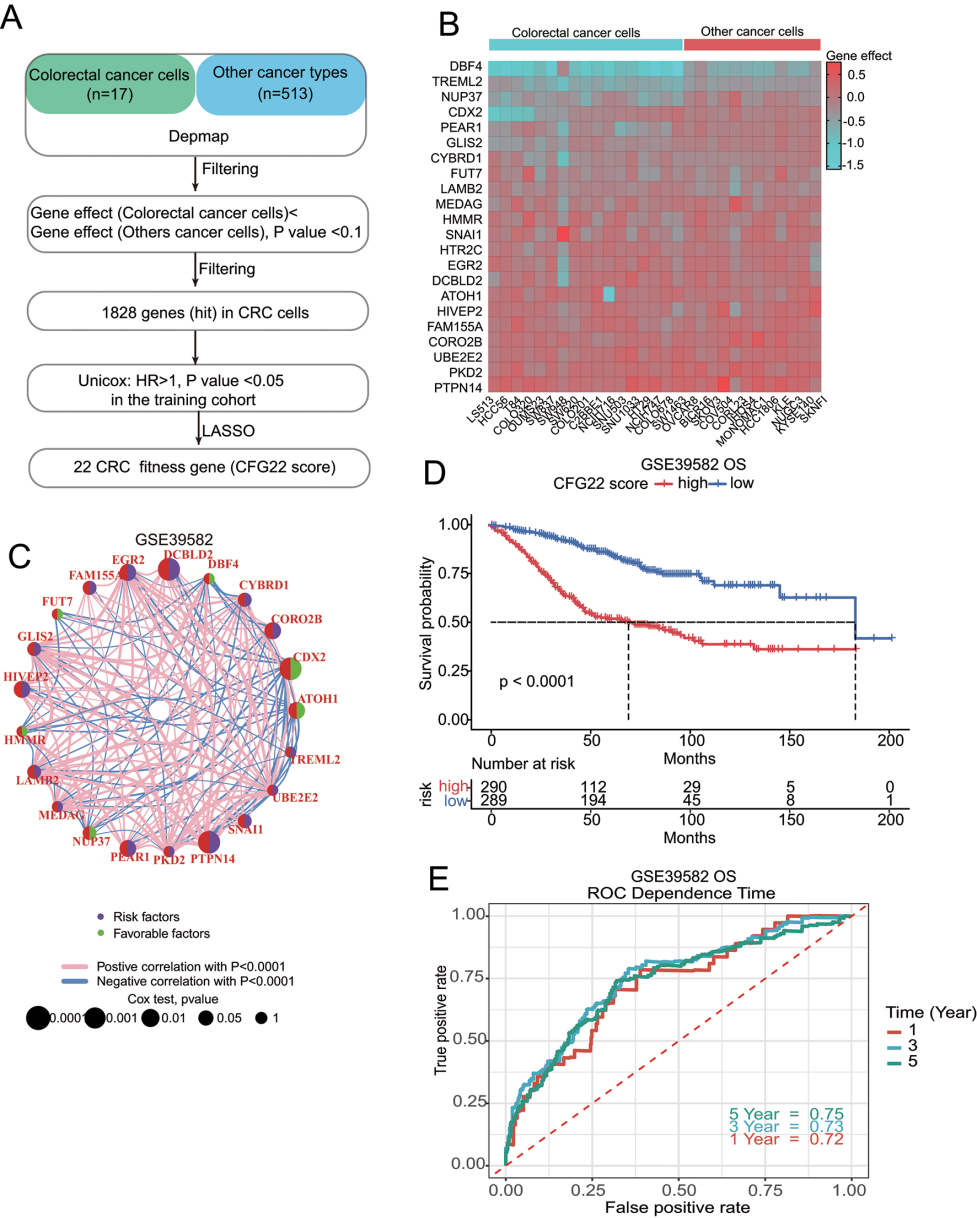
Analysis of specific cell-dependent genes in colorectal cancer and identification of a 22-gene prognostic signature. (**A**) Flow diagram of identifying the 22-gene colorectal cancer (CRC) fitness gene signature (CFG22 score). (**B**) Heat map shows gene effects of 22 genes identified in the prognostic signature. (**C**). The correlation between 22 genes and univariate test of each gene in the GSE39582 cohort. (**D**) Kaplan-Meier assessment of overall survival according to the CFG22 score in GSE39582 cohort. (**E**) One, three, five-year receiver operator characteristic (ROC) curves of overall survival for GSE39582 cohort. OS, overall survival

To find the specific dependency genes associated with the proliferation of CRC cells, we extensively investigated genes between 17 CRC cells and 513 other cancer type cells and found 1828 genes that played relatively important roles in the proliferation of CRC cells. By univariate cox analysis, 113 of these 1828 genes were significantly correlated with prognosis. Through the Lasso algorithm of the 113 genes, we defined the CFG22 score model (Fig. 1A). Heatmap shows differences in gene dependency of the selected 22 genes for the CRC cells and other cancer type cells in the model (Fig. 1B). Among these genes, DBF4, TREML2, and NUP37 might be key to CRC cells proliferation (Supplementary Fig. 1A). Analysis of correlation among the expression of these genes showed the genes were closely related to each other (Fig. 1C and Supplementary Fig. 1B). Kaplan–Meier analysis showed that the CFG22 score had a preferable ability to predict the prognosis of CRC patients, and patients with high CFG22 score had inferior overall survival in the GSE39582 cohort (Fig. 1D). Time-dependent receiver operator characteristic (ROC) analysis showed with the increase of time, the CFG22 score model had better predictive accuracy (Fig. 1E).

### The model embodied the robustness of prediction efficiency

**Fig. 2:**
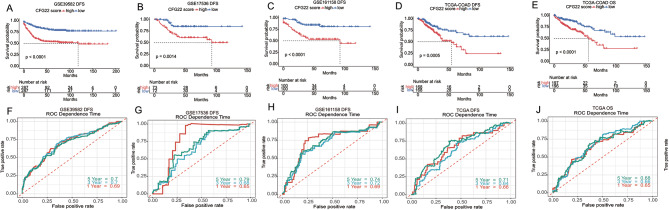
The CFG22 score is associated with overall survival and disease free survival in patients with colorectal cancer. (**A**-**D**) Kaplan-Meier evaluation of disease free survival (DFS) according to the CFG22 score in the GSE39582 (**A**), GSE17536 (**B**), GSE161158 (**C**), TCGA (**D**) cohorts. (**E**) Kaplan-Meier evaluation of overall survival (OS) according to the CFG22 score in TCGA cohorts. (**F**-**J**) Time-dependent receiver operator characteristic (ROC) analysis for predicting DFS at one, three, five-year in the GSE39582 (**F**), GSE17536 (**G**), GSE161158 (**H**), TCGA (**I**) cohorts and OS in TCGA (**J**) cohorts. High- and low-risk group was identified according to the median of CFG22 score

We comprehensively evaluated the CFG22 score model’s ability to predict overall survival and disease free survival in other independent cohorts from microarray platforms and the Illumina RNA-seq platform. In the microarray platforms GSE39582, GSE17536, and GSE161158, high CFG22 score was significantly correlated with poor disease free survival (Fig. 2A–2C). In the Illumina RNA-seq platform TCGA-COAD, the CFG22 score still had a good performance to predict the both disease free survival (Fig. 2D) and overall survival (Fig. 2E), representing the robustness of prediction efficiency of the CFG score model. In the independent cohorts of both microarray platforms and the Illumina RNA-seq platform, time-dependent ROC analysis showed with the increase of time, the CFG22 score model also hold better predictive accuracy (Fig. 2F–2J).

### Genomic abnormalities of the genes in CFG22 score model and subgroup

**Fig. 3:**
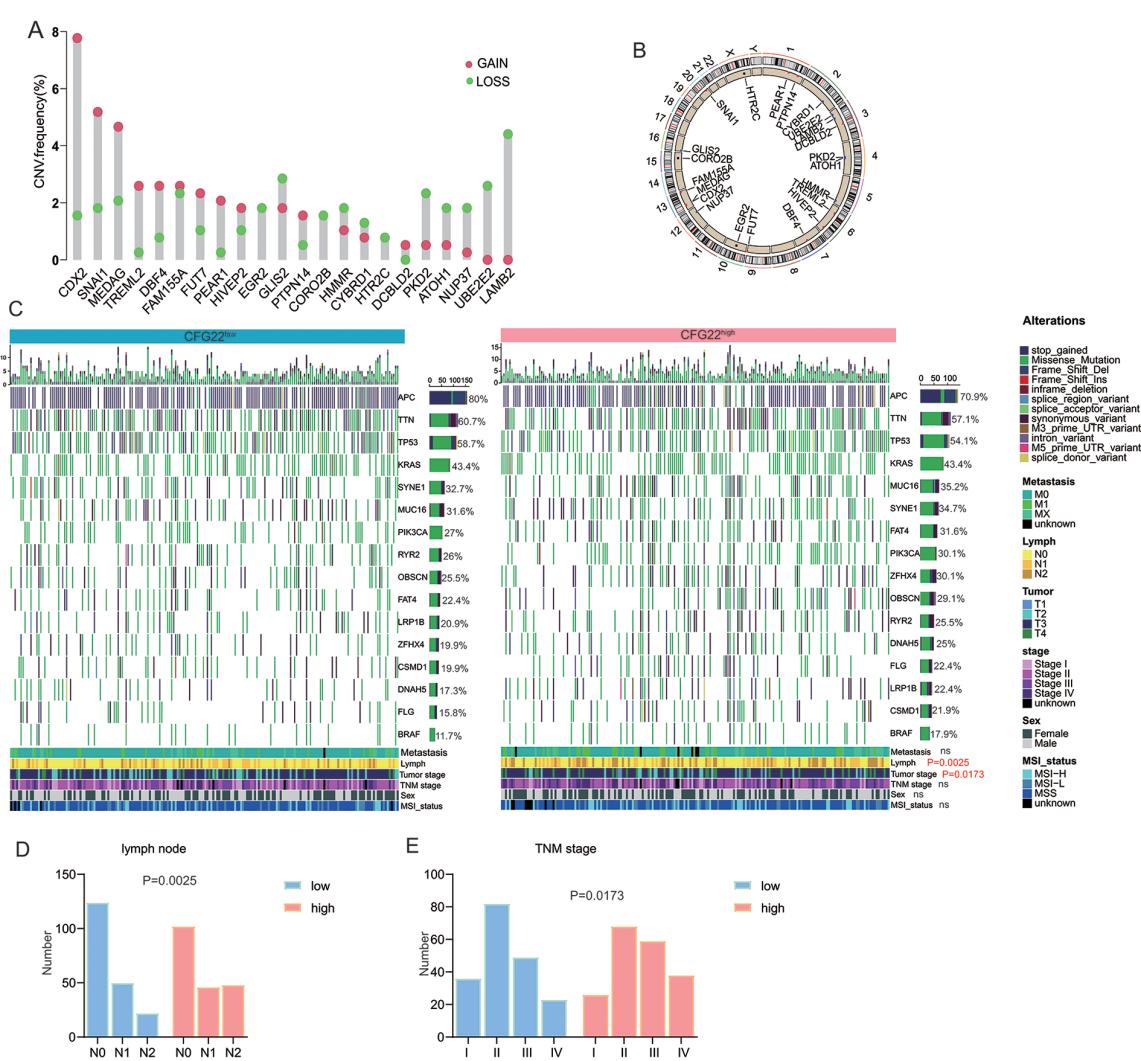
Genomic abnormalities of the genes in CFG22 score model (**A**) Frequency of copy number variation (CNV) of 22 gene identified in the CFG22 score model. (**B**) Circos map shows genes located in chromosome segments. (**C**) Heatmap shows somatic mutations and clinical information between CFG22 high and CFG22 low patient groups. (**D**-**E**) Histogram showed the number of each stage of lymph node and TNM staging. Ns, not significant

We systematically analyzed copy number variation (CNV) frequency types of gain and loss of the 22 genes involved in the model in the TCGA cohort. The CNV frequency of gain is higher than loss of 11 genes (CDX2, SNAI1, MEDAG, TREML2, DBF4, FAM155A, FUT7, PEAR1, HIVEP2, PTPN14, and DCBLD2). EGR2, CORO2B, and HTR2C were in terms of the same frequency of gain and loss. And 8 genes (GLIS2, HMMR, CYBRD1, PKD2, ATOH1, NUP37, UBE2E2, and LAMB2) of the model had a higher frequency of loss than gain (Fig. 3A). They were dispersed on the most chromosomes (Fig. 3B). Some mutations and clinicopathologic features were associated with outcome in CRC, thus, we investigated these variates between CFG22^high^ and CFG22^low^ group. In the CFG22^high^ group, the frequency of FAT4, ZFHX4, FLG and BRAF were higher than these in the low group. For lymph node and TNM stage, patients in the CFG22^high^ group had higher frequency of N2 stage and TNM III and IV stage (Fig. 3C and E). Besides, we investigated the predictive value of the mutation of BRAF, KRAS, and TP53 in the GSE39582 and TCGA cohorts. Results showed that the mutation of BRAF, KRAS, and TP53 gene alone can only weakly or not predict the prognosis of CRC patients (Supplementary Fig. 2A-2 F), which suggested the need for other more effective predictors.

### Construction of the nomogram model

**Fig. 4:**
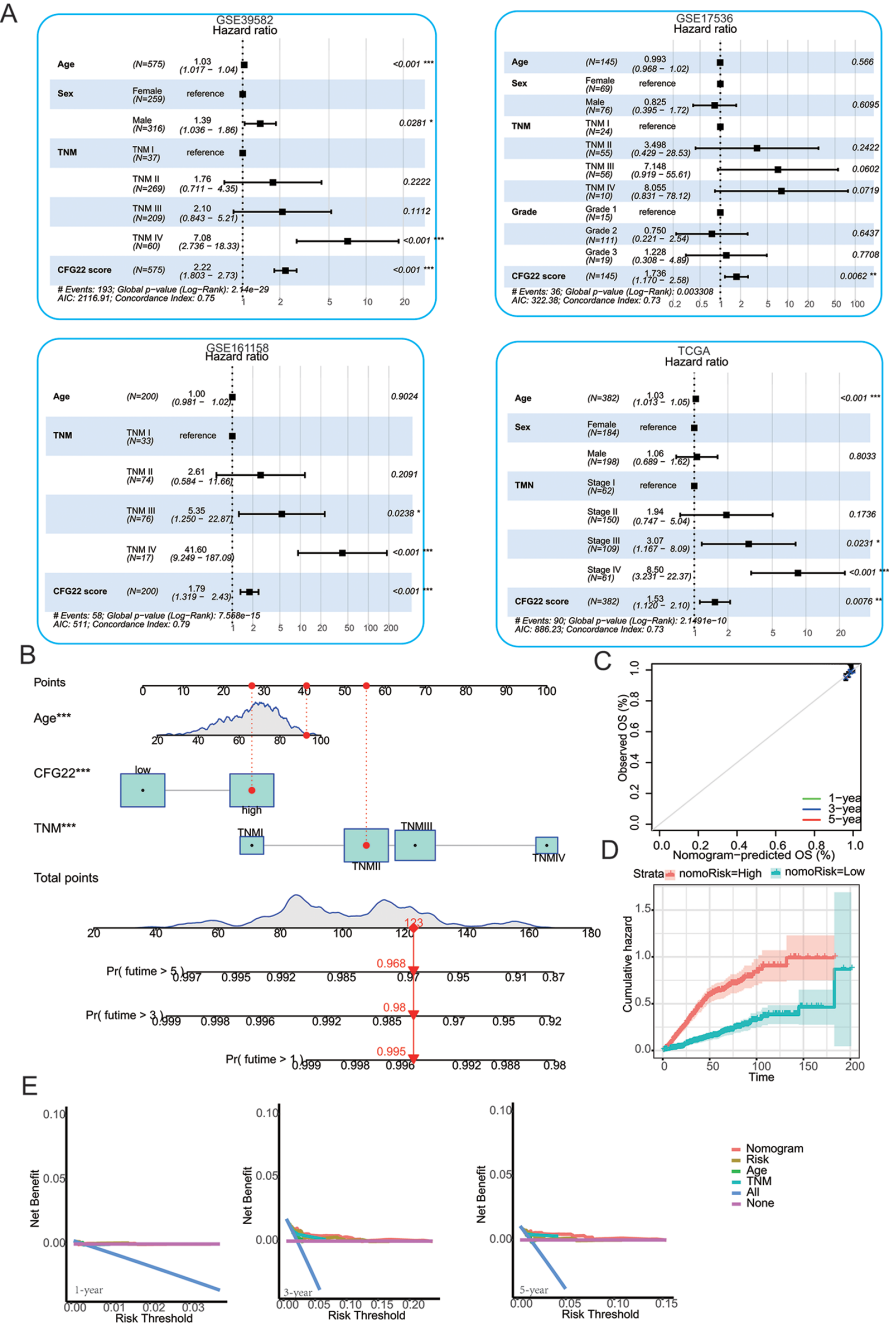
Construction of the nomogram model (**A**) Multivariate cox regression analysis of the CFG22 score and clinicopathologic features in the GSE39582, GSE17536, GSE161158, TCGA cohorts. (**B**-**D**) The nomogram prediction model for the probability of survival (**B**), calibration curves (**C**) and cumulative hazard (**D**) in patients with of colorectal cancer in the merged cohort containing the GSE39582, GSE17536, GSE161158, TCGA cohorts. (**E**) Decision curve analysis in the merged cohorts. X-axis represents risk threshold and y-axis represents net benefit. High- and low-risk group was identified according to the median of CFG22 score. *, *p* < 0.05; **, *p* < 0.01; ***, *p* < 0.001

We performed the multivariate cox regression analysis of the CFG22 score and clinicopathologic features in the GSE39582, GSE17536, GSE161158, TCGA cohorts. Results showed CFG22 score was an independent risk factor for the patients with CRC (Fig. 4A). Then we constructed a clinical prediction nomogram model in the merged cohort according to variates age, TNM stage, CFG22 risk stratification. For each variate, they were assigned values ranging from 0 to 100. The values for each variate add up to give a total point, which could provide guidance for prognosis (Fig. 4B–4D). Besides, the decision curve analysis showed the nomogram had a preferable clinical net benefit (Fig. 4E).

### Infiltrating immune cells in the CFG22 risk stratification

**Fig. 5:**
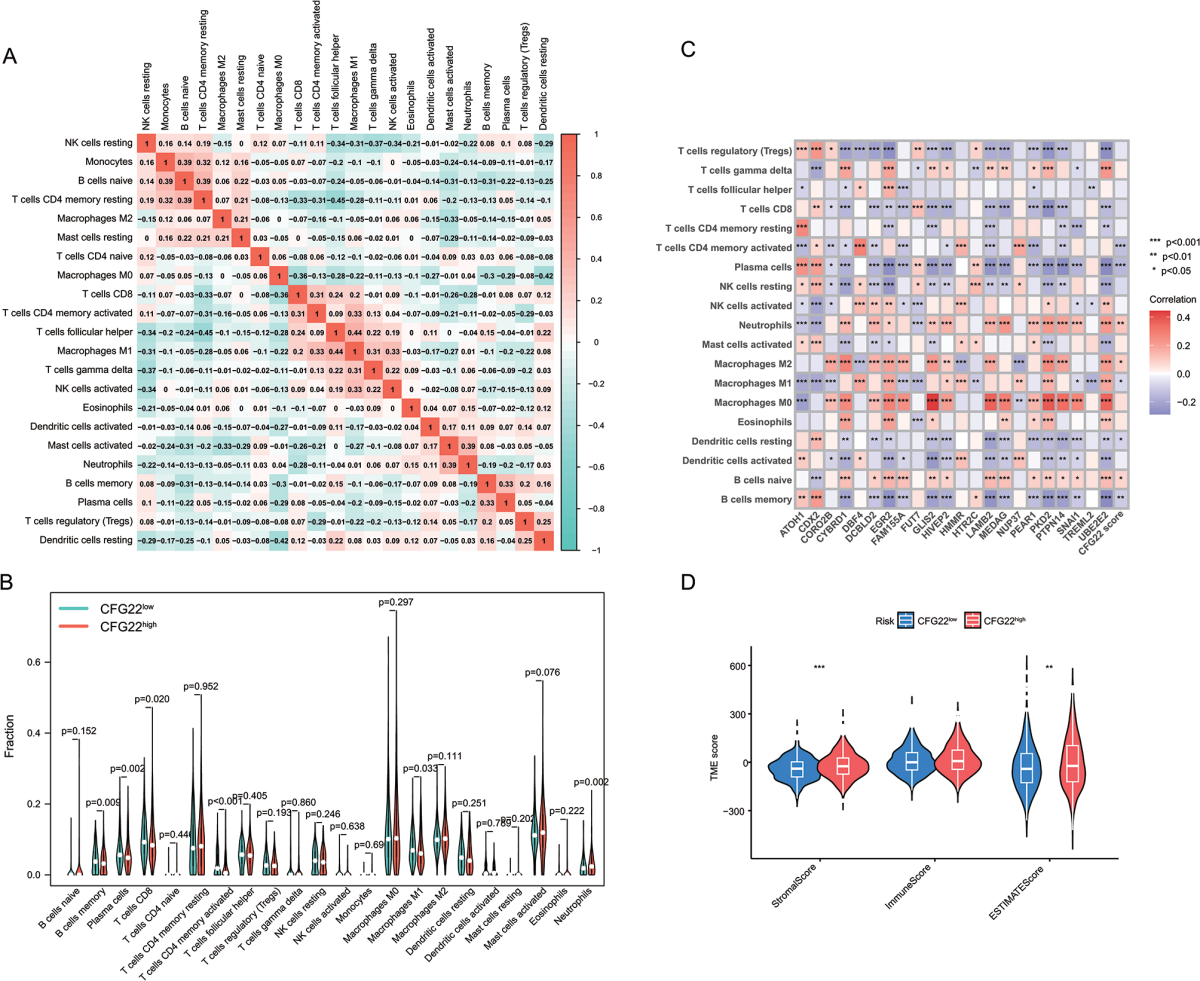
Immune cells infiltration between the CFG22^low^ and CFG22^high^ group. (**A**) Correlation analysis of infiltrating immune cells in the model. (**B**) The differential distribution of immune cells between the CFG22^low^ and CFG22^high^ group. (**C**) Correlation analysis between infiltrating immune cells and CFG22 score or gene expression. (**D**) Variation analysis of stromal score, immune score, and ESTIMATE score between the CFG22^low^ and CFG22^high^ group. *, *p* < 0.05; **, *p* < 0.01; ***, *p* < 0.001

To explore the infiltration of immune cells between the CFG22^high^ and CFG22^low^ groups, we systematically analyzed correlation of infiltration fraction of different immune cells. Results showed the fraction of T cells follicular helper was significantly positive correlated with the fraction of macrophages M1. While the fraction of T cells follicular helper was obviously negative correlated with the fraction of T cells CD4 memory resting (Fig. 5A). Compared with in the CFG22^low^ group, patients in the CFG22^high^ group showed lower infiltration of B cell memory (*p* = 0.009), plasma cells (*p* = 0.002), T cells CD8+ (*p* = 0.020), T cells CD4 + memory activated (*p* < 0.001), macrophages M1 (*p* = 0.033), but higher infiltration of neutrophils (*p* = 0.002) (Fig. 5B). For correlation analysis between the CFG22 score and the infiltration of immune cells, the CFG22 score was positive related with the fraction of neutrophils, macrophages M2, and B cells naïve. While the score was in the remarkably negative correlation with the fraction of T cells CD4 + memory activated, plasma cells, macrophages M1, dendritic cells resting, and B cells memory (Fig. 5C). For tumor microenvironment analysis, we found patients in the CFG22^high^ showed higher stromal score and ESTIMATE score than these in the CFG22^low^ group (Fig. 5D).

### Comparisons of the CFG22 score model and other gene expression signatures

**Fig. 6:**
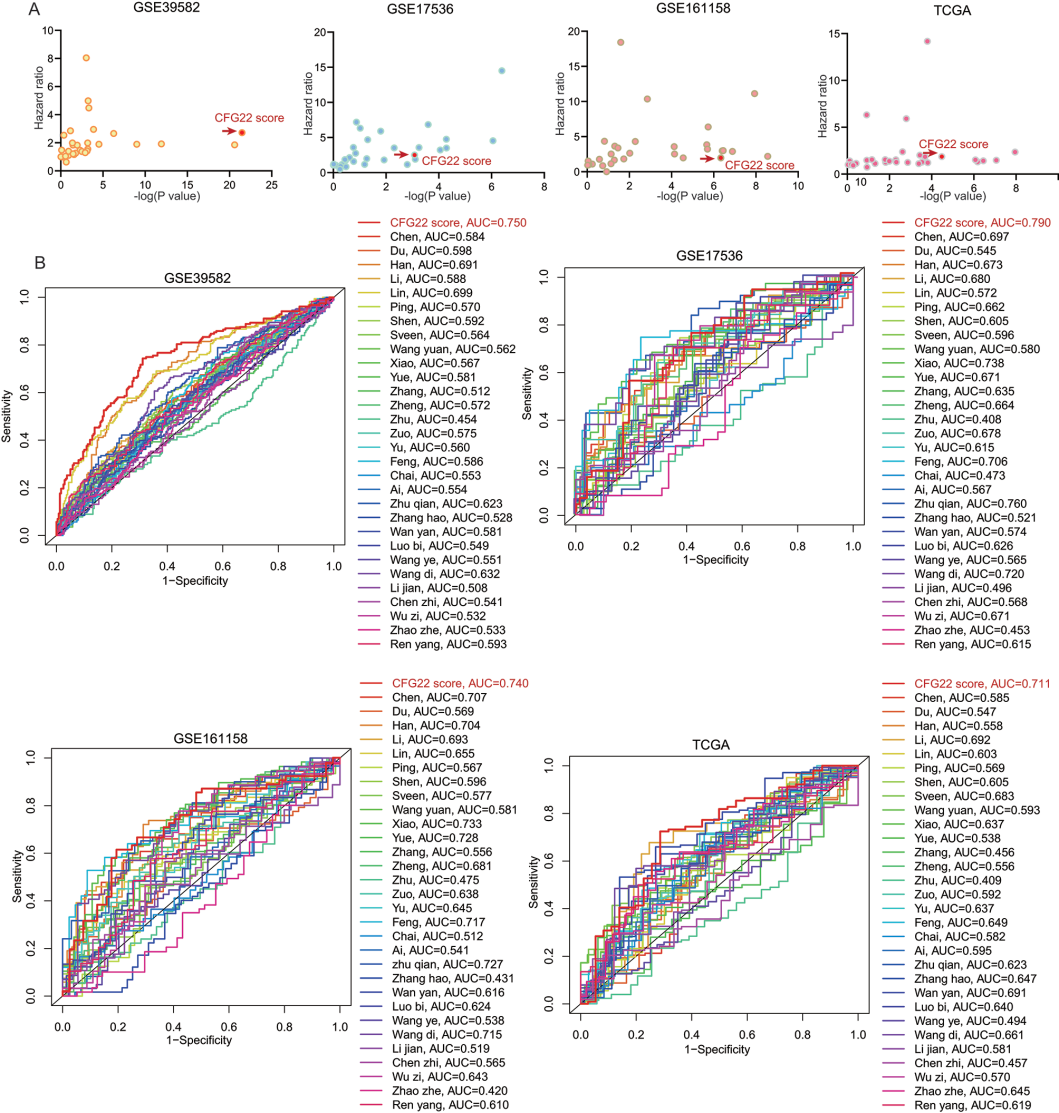
Comparisons of CFG22 score model and other gene expression signatures. (**A**) Univariate cox regression analysis of the CFG22 score and other 30-published signatures. (**B**) Receiver operator characteristic (ROC) analysis for the CFG22 score and other 30-published signatures in the GSE39582, GSE17536, GSE161158, TCGA cohorts

To compare the prognostic performance of the CFG22 model with other signatures, we systematically investigated 30-published signatures, which were all mRNA signatures built by lasso algorithms. By univariate cox regression analysis of the CFG22 score and other 30-published signatures, we found the CFG22 score were all significantly associated with poor survival in the GSE39582, GSE17536, GSE161158, and TCGA cohorts (Fig. 6A). For each signature, we also evaluated area under curve (AUC). Result showed the CFG22 score model was remarkably ranked first in predictive performance in the GSE39582, GSE17536, GSE161158, and TCGA cohorts, which had a better possibility of extrapolation for other platforms and institutions (Fig. 6B).

### The CFG22 score provided a promising treatment strategy for CRC

**Fig. 7:**
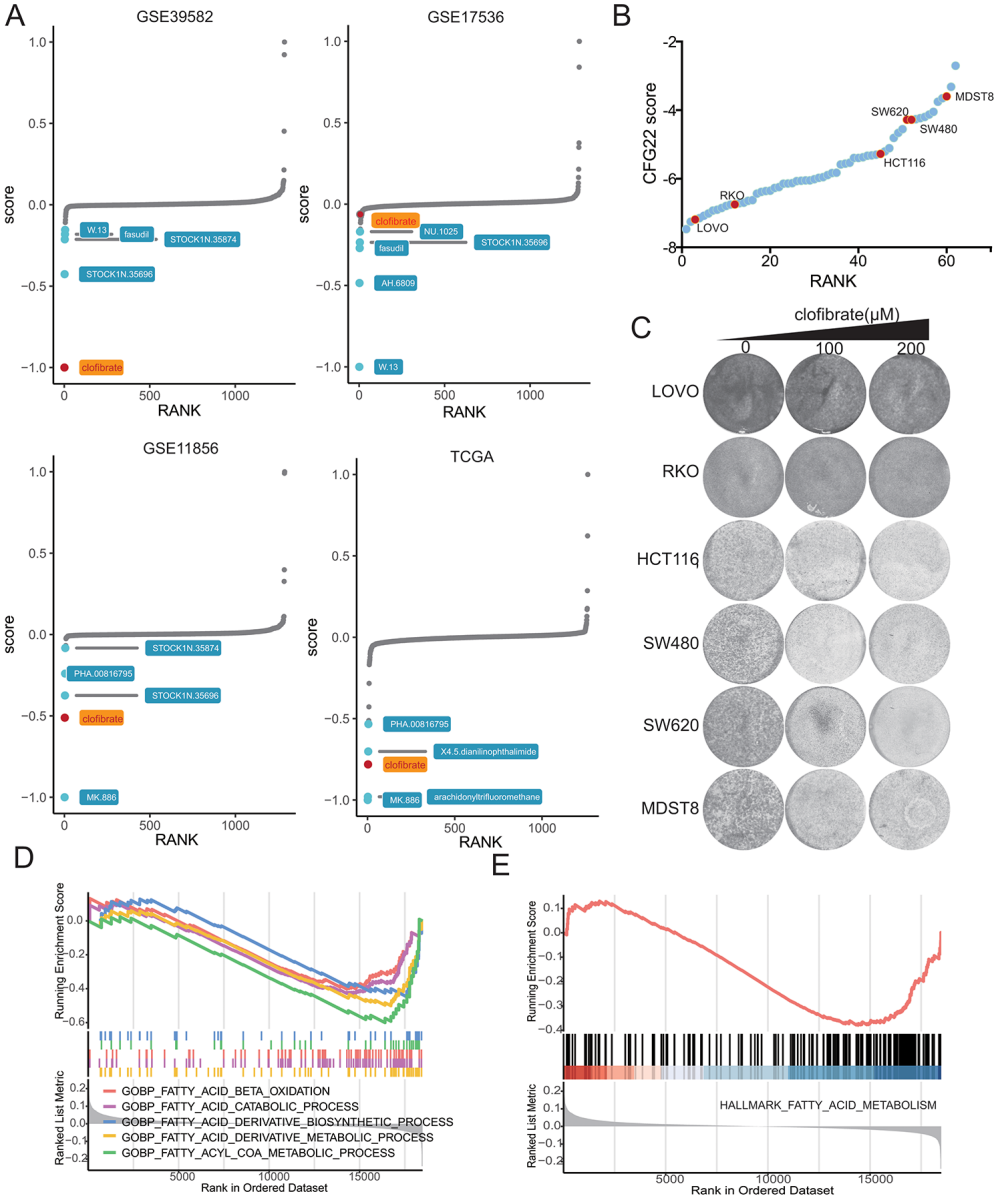
Drug sensitivity analysis between the CFG22^low^ and CFG22^high^ group. (**A**) The connectivity map (CMap) analysis predicts the priority of drug of high-risk group. High- and low-risk group was identified according to the median of CFG22 score. (**B**) The CFG22 score of 62 colorectal cancer (CRC) cells. (**C**) Long-term proliferation assay shows drug susceptibility of clofibrate in different CRC cells. (**D**-**E**) Gene set enrichment analysis of biological process (**D**) and hallmark (**E**) of fatty acid metabolic process

Given the prominent association between the high CFG22 score and poorer patient outcomes, we studied whether patients with the high CFG22 score could benefit from other treatment strategies besides chemotherapy. By an optimal approach for LINCS data-based therapeutic discovery, we found clofibrate ranked top and might be a potential treatment for patients with the high CFG22 score (Fig. 7A). To test the reliability of this prediction, we analyzed the CFG22 score of 62 CRC cells (Fig. 7B). According to its rank of the CFG22 score, we performed long-term proliferation assay of LOVO, RKO, HCT116, SW480, SW620 and MDST8 cells with the treatment of clofibrate. Results showed CRC cells with high CFG22 score seemed to be more sensitive to clofibrate, and LOVO and RKO cells were insensitive to the drug (Fig. 7C). Gene set enrichment analysis of biological process and hallmark pathways showed patients in the CFG22^high^ group presented with disorder of fatty acid metabolism, which showed the possible mechanism of the treatment of clofibrate (Fig. 7D and E).

## Discussion

In recent years, a growing number of researchers have explored the prognostic factors of CRC, and many new CRC risk stratification prediction models have emerged, most of which rely on TNM staging and colorectal examination. Although TNM staging provides a reliable survival rate, there are significant difference in survival times among patients at the same stage who receive similar treatments, which indicates that TNM staging does not accurately predict the prognosis of CRC patients [11]. Currently recognized as a groundbreaking technology for gene editing, CRISPR-Cas9 has many advantages such as high editing efficiency, ease of use, and low cost. It has been widely used in fields such as genetic diseases, infectious diseases, and tumors, providing a promising solution for the treatment of various diseases [25]. Wang et al. identified cancer essential genes (CEGs) with prognostic value through CRISPR-Cas9 screening, established and validated three distinct subtypes of pancreatic cancer (PC) patients in a multicenter study [26]. These findings not only deepen our understanding of PC molecular heterogeneity but also address the clinical need for risk stratification and personalized treatment in the era of precision medicine.

Currently, CRISPR-Cas9 gene editing technology stands as the most powerful tool for regulating gene expression and is extensively utilized in high-throughput functional screening to strengthen the specific genetic background of key genes essential for human cell growth and proliferation, particularly in the pathogenesis and drug resistance mechanisms of tumors [27]. Ouyang et al. employed CRISPR-Cas9 technology to screen for novel genes associated with cisplatin resistance in ovarian cancer cell lines using the GeCKO library [28]. Following CRISPR-Cas9-mediated knockout of relevant genes and identification of predictive markers, ZNF587B and SULF1 were found to be associated with cisplatin resistance. Notably, ZNF587B emerged as a potential risk stratification biomarker for predicting cisplatin resistance. CRC is characterized by a complex interplay of genetic and epigenetic alterations. CRISPR-Cas9 gene editing has emerged as a powerful tool for epigenetic modification and has been implicated in the development and progression of various cancers and diseases. Its remarkable mutation efficiency, simplicity, and affordability make CRISPR-Cas9 a promising targeted genome modification technology with wide-ranging applications [29].

Colorectal cancer has the characteristics of slow and gradual development, symptoms that are not typical of colorectal cancer, rapid progression, a high ability to spread to other parts of the body, and a low chance of survival. Clinically, there is an urgent need for tumor markers with high sensitivity and specificity to detect and diagnose CRC early and improve patient outcomes [30]. This study refers to observing the expression and prognosis of DBF4, TREML2 or NUP37 in cancer, and exploring its function, clinical significance, prognostic value and possible mechanism in colorectal cancer, providing possibilities for the treatment of colon cancer. In recent years, studies by Matthews LA and others have proven that the expression of DBF4 is low in normal tissues, but its expression is abnormally elevated in a variety of cancers [31]. Nambiar S et al. found that DBF4, as a molecular determinant related to prognosis, has increased expression in melanoma cells and confers a proliferation advantage [32]. Researchers such as Qi found that the expression of DBF4 is up-regulated in lung cancer, promoting tumor growth and invasion [33]. Wang et al. have demonstrated that elevated DBF4 expression is associated with gastric cancer progression, invasiveness, and resistance to 5-Fu chemotherapy [34]. Wang et al. confirmed for the first time that TREML2 regulates inflammation by regulating microglial polarization and NLRP3 inflammasome, revealing the mechanism of TREML2 regulating microglia, suggesting that TREML2 inhibition is a new direction for AD treatment [35]. Li et al. reported that high expression of NUP37 can promote the proliferation and migration of breast cancer cells. Knocking out NUP37 has an inhibitory effect on the proliferation, migration, epithelial-mesenchymal transition and stem cells of breast cancer cells, suggesting that NUP37 has a tumorigenic role in the biological characteristics of breast cancer cells [36]. Zhang et al. reported that NUP37 is highly expressed in gastric cancer cells and tissues [37]. NUP37 activates (phosphatidylinositol3-kinase/Protein kinase B/mammalian target of rapamycin, PI3K/AKT/mTOR) signaling pathway promotes the proliferation, invasion and migration of various gastric cancer cell lines, inhibits cell apoptosis, and plays a role as a tumor activator in cancer cells. By reviewing the literature, we found that there are currently few reports on the research on DBF4, TREML2 or NUP37 in colorectal cancer. Therefore, it is confirmed that DBF4, TREML2 or NUP37 may play a key role in the occurrence and development of colorectal cancer, manifesting as cancer. The cell biological properties of genes have important research value. Its clinical significance deserves further exploration.

Upon analyzing the immune infiltration of the high-risk group and the low-risk group, Plasma cells, CD8 + T cells, CD4 + memory T cells, and macrophages M1 were significantly reduced, while neutrophils were observably increased in the CFG22 high group. We speculate that dynamic changes in neutrophils and lymphocytes can reflect the balance between the inflammatory response and the immune system, and may reflect the treatment effect and prognosis of colorectal malignant tumors. Tumor infiltrating lymphocytes (TILs) are a heterogeneous lymphocyte population dominated by T cells that reside in the tumor microenvironment. They play an important role in the TME and have emerged as promising prognostic indicators for various cancers. In primary CRC, TILs are reliable prognostic indicators and outperform TNM staging in disease assessment [38]. Tumor-associated neutrophils (TANs) have been implicated in the regulation of CRC development and progression, with implications for prognosis. Wu et al. found that TANs infiltration was associated with poorer overall survival compared to negative TANs infiltration [39]. This may be due to TANs infiltration fostering a pro-inflammatory tumor microenvironment that promotes cancer cell proliferation. Additionally, TANs infiltration may induce cancer cell metastasis to surrounding lymph nodes and distant sites, contributing to accelerated disease progression and poor prognosis. Studies have demonstrated that TANs can regulate tumor development via interleukin-17a secretion. RAZI S et al. found that IL-17a, a key member of the IL-17 family, exerts pro-inflammatory effects primarily by promoting the release of inflammatory mediators and recruiting inflammation-related cells [40]. Activation of IL-17a can promote tumor proliferation and angiogenesis. Tumor metastasis is the leading cause of death in patients with solid tumors. Circulating tumor cells (CTCs) are tumor cells that detach from the primary tumor and enter the bloodstream. They are considered the “seeds” of tumor metastasis. Research by SZCZERBA et al. also found that CTCs in CTC-neutrophil clusters exhibit higher proliferation and viability compared to single CTCs, and several clinical studies have confirmed the association between CTC-neutrophil clusters and poor prognosis in cancer patients [41]. XUE P et al. demonstrated that the neutrophil-to-lymphocyte ratio (NLR) can also serve as an indicator for prognostic evaluation of pancreatic cancer patients undergoing chemotherapy [42]. For patients with advanced pancreatic cancer who underwent chemotherapy, a greater reduction in NLR value, longer treatment duration, and higher overall survival rate were observed after one cycle of treatment compared to patients with localized pancreatic cancer. Patients with NLR > 5 exhibited higher levels. These findings suggest that NLR can serve as a prognostic indicator for patients with advanced pancreatic cancer receiving chemotherapy. The development and progression of tumors are often associated with inflammatory responses, which can also influence the prognosis of cancer patients. Peripheral blood cell count is a routine and cost-effective clinical examination method. Utilizing NLR as a reference indicator to assess the prognosis of patients with malignant tumors will not impose additional financial or emotional burdens on patients. As a relatively sensitive inflammatory response marker, NLR can aid clinicians in early evaluation of tumor severity and prognosis.

Due to the complexity of the molecules of CRC, there are considerable obstacles to clinical implementation of risk stratification systems, such as TNM staging [43]. Only a limited set of molecular signatures have been used to guide the treatment of advanced CRC [44, 45]. Clinically available transcriptomic techniques are characterized by repeatability and analytical effectiveness, and have great potential to reveal prognostic transcriptome information for CRC, which will allow rapid risk assessment for all patients indiscriminately [46]. We constructed the CFG22 score model composed of 22 fitness genes screened by genome-scale CRISPR-Cas9 knockout screens, which mainly reflected the essentially molecular and clinically relevant characteristics of the fitness of CRC cells. The molecular classification of CRC describes tumor heterogeneity based on gene expression patterns and contributes to understanding the biology of tumor formation, growth, and prognosis. We compared the CFG22 score model with currently published 30 risk stratification schemes and found that the CFG22 score better stratified and predicted prognosis, helping to reassign patients’ risk at diagnosis for more appropriate treatment, which might provide a powerful auxiliary prediction tool for TNM staging. Our model is based on the inherent vulnerabilities of colon cancer and is well validated in external cohorts. Genes we identified by genome-scale CRISPR-Cas9 knockout screens associated with proliferation of CRC cells, for instance, DCBLD2, EGR2, HMMR, etc. may be involved in biological processes that make tumor cells more malignant or more likely to evade chemotherapy, enhancing their adaptability [47–49]. Of course, we still need more external independent cohorts to verify and provide the possibility for further promotion of the model. And knockdown gene experiments or knock-out experiments need to be performed to further confirm the relationship between the discovered genes and proliferation of CRC cells.

In this study, using drug analysis and pathway enrichment analysis, we found that the high-risk group of colon cancer may be more sensitive to clofibrate drugs. In recent years, Chen et al. screened out clofibrate, which reduced Homologous repair deficiency (HRD) scores to improve oncological outcomes in breast cancer and help develop personalized clinical management and treatment options for breast cancer [50]. Xue et al. demonstrated that PPARα is overexpressed in pancreatic cancer tissue, and clofibrate- mediated PPARα activation sensitizes pancreatic cancer cells to radiation via the Wnt/β-catenin pathway [51]. Karthic Chandran et al. demonstrated that PPARα down-regulates inflammation and adipogenesis pathways through activation of its agonist clofibrate, while inhibiting the growth of human breast cancer cells [52]. These findings provide new insights into our understanding of the role of clofibrate drugs in cancer treatment and support the use of PPARα agonists as therapeutic anticancer agents.

## Conclusion

In general, we analyzed key cancer dependencies of CRC based on genome-scale genes of CRC, which were key candidates for the treatment of CRC and deserved further exploration to determine their therapeutic significances, such as, DBF4, TREML2 or NUP37, etc. Besides, the model we obtained enables rapid risk assessment of newly diagnosed colon cancer patients. We have also proposed a new clofibrate drug -based strategy to treat high-risk CRC and have tentatively identified biomarkers of drug sensitivity.

### Electronic supplementary material

Below is the link to the electronic supplementary material.

Supplementary Material 1

Supplementary Material 2

Supplementary Material 3

## Data Availability

The raw experimental data and analysis codes supporting the conclusions of this article will be made available by the corresponding authors.
