# Supplementary material for: Genome wide-scale CRISPR-Cas9 knockout screens identify a fitness score for optimized risk stratification in colorectal cancer
Source: J Transl Med. 2024 Jun 10;22:554. doi: 10.1186/s12967-024-05323-3 (PMC11163718; doi:10.1186/s12967-024-05323-3)
Supplement: Supplementary file 3 — Supplementary Material 3 [file 12967_2024_5323_MOESM3_ESM.docx]

Supplementary Figure 1. (A) Density distribution of gene effect of DBF4, TREML2, NUP37 compared CRC cells and other 513 cancer cells. (B) The correlation between 22 genes and univariate test of each gene in the GSE17536, GSE161158, TCGA cohorts.

Supplementary Figure 2. Kaplan–Meier evaluation of overall survival and disease free survival according to the status of BRAF (A-B), KRAS (C-D), and TP53 (E-F) mutation in the GSE39582 (A, C, E) and TCGA (B, D, F) cohorts.
